# Prenatal alcohol exposure and childhood atopic disease: A Mendelian randomization approach^☆^

**DOI:** 10.1016/j.jaci.2013.04.051

**Published:** 2014-01

**Authors:** Seif O. Shaheen, Clare Rutterford, Luisa Zuccolo, Susan M. Ring, George Davey Smith, John W. Holloway, A. John Henderson

**Affiliations:** a Centre for Primary Care and Public Health, Barts and The London School of Medicine and Dentistry, London, United Kingdom; b School of Social and Community Medicine, University of Bristol, Bristol, United Kingdom; c Medical Research Council Centre for Causal Analyses in Translational Epidemiology, University of Bristol, Bristol, United Kingdom; d Human Development and Health, Faculty of Medicine, University of Southampton, Southampton, United Kingdom

**Keywords:** Alcohol, ADH1B, Mendelian randomization, prenatal exposure, ALSPAC, pregnancy, birth cohort, asthma, atopy, ADH, Alcohol dehydrogenase, ALSPAC, Avon Longitudinal Study of Parents and Children (ALSPAC), GWAS, Genome Wide Association Study, PCA, Principal Components Analysis

## Abstract

**Background:**

Alcohol consumption in western pregnant women is not uncommon and could be a risk factor for childhood atopic disease. However, reported alcohol intake may be unreliable, and associations are likely to be confounded.

**Objective:**

We aimed to study the relation between prenatal alcohol exposure and atopic phenotypes in a large population-based birth cohort with the use of a Mendelian randomization approach to minimize bias and confounding.

**Methods:**

In white mothers and children in the Avon Longitudinal Study of Parents and Children (ALSPAC) we first analyzed associations between reported maternal alcohol consumption during pregnancy and atopic outcomes in the offspring measured at 7 years of age (asthma, wheezing, hay fever, eczema, atopy, and total IgE). We then analyzed the relation of maternal alcohol dehydrogenase *(ADH)1B* genotype (rs1229984) with these outcomes (the A allele is associated with faster metabolism and reduced alcohol consumption and, among drinkers, would be expected to reduce fetal exposure to ethanol).

**Results:**

After controlling for confounders, reported maternal drinking in late pregnancy was negatively associated with childhood asthma and hay fever (adjusted odds ratio [OR] per category increase in intake: 0.91 [95% CI, 0.82-1.01] and 0.87 [95% CI, 0.78-0.98], respectively). However, maternal *ADH1B* genotype was not associated with asthma comparing carriers of A allele with persons homozygous for G allele (OR, 0.98 [95% CI, 0.66-1.47]) or hay fever (OR, 1.11 [95% CI, 0.71-1.72]), nor with any other atopic outcome.

**Conclusion:**

We have found no evidence to suggest that prenatal alcohol exposure increases the risk of asthma or atopy in childhood.

Experiments in animals have shown that short-term alcohol exposure inhibits T_H_1 responses and increases T_H_2 cytokine responses and IgE,^1^, ^2^ although longer-term consumption has been shown to reduce airway hyperresponsiveness and allergic inflammation in an adult mouse model.^3^ In addition, prenatal exposure can impair fetal lung growth and development,^4^, ^5^, ^6^ through increased oxidative stress.^7^ In humans, reported alcohol intake is positively associated with total IgE cross-sectionally, although associations with allergen-specific sensitization have been inconsistent.^8^, ^9^, ^10^ Studies from Denmark have found that reported alcohol consumption in pregnancy was positively associated with total IgE in cord blood^11^ and postnatal atopic dermatitis^12^, ^13^ but not with hospitalization for asthma in childhood.^14^ Given these data, and that the prevalence of low-to-moderate alcohol consumption during pregnancy is high (30%-59%) in the United Kingdom, Denmark, Sweden, Australia, and the United States,^15^, ^16^, ^17^, ^18^, ^19^, ^20^ it would seem important to establish whether prenatal alcohol exposure might be contributing to the high prevalence of childhood atopic disease in the west.^21^

One difficulty with using reported alcohol intake in pregnancy to measure exposure is that underreporting of consumption is common^22^ and, as with all observational studies, associations may arise spuriously through bias, or because of unmeasured or residual confounding, a particular problem in nutritional epidemiology.^23^ Randomized trials of alcohol exposure in pregnancy would be unethical, and we are not aware of any trials of alcohol avoidance in pregnancy which have evaluated childhood atopic outcomes. However, causal inference can be strengthened in observational studies with the use of Mendelian randomization to reduce measurement error and to minimize bias and confounding.^24^, ^25^ This approach hinges on the principle that genotype is randomly allocated during meiosis, and consequently associations between genetic variants and disease are not generally susceptible to confounding by lifestyle factors. In addition, variants should not be associated with the disease outcome of interest except through their link to the modifiable risk process of interest. In the case of alcohol, a nonsynonymous variant in the alcohol metabolizing gene, alcohol dehydrogenase *(ADH)1B*, is thought to accelerate ethanol clearance and to inhibit heavy drinking^26^ and can be used as a proxy for alcohol consumption. The A allele is associated with faster conversion of alcohol to acetaldehyde, and this leads to reduced alcohol consumption. In European populations, this *ADH1B* variant is associated with aerodigestive cancers,^27^ thus providing convincing evidence that alcohol causes these conditions. Furthermore, in the Avon Longitudinal Study of Parents and Children (ALSPAC), a United Kingdom population-based birth cohort, the maternal *ADH1B* gene variant was strongly associated with alcohol use before and during pregnancy, suggesting that it could be used as a reliable instrument to study the consequences of prenatal (and periconceptional) alcohol exposure.^28^ Indeed, to study the relation between fetal alcohol exposure and cognitive development, we recently used a Mendelian randomization approach in ALSPAC to determine whether genetic variants in alcohol metabolizing genes were associated with childhood IQ.^29^

In ALSPAC, we have first analyzed whether reported alcohol intake by mothers in pregnancy is associated with risk of atopic disease in the offspring and then examined the relation between the maternal *ADH1B* gene variant and these outcomes.

## Methods

### Subjects

ALSPAC is a population-based birth cohort that recruited 14,541 predominantly white pregnant women resident in the Bristol area of the United Kingdom during 1990 to 1992. There were 14,062 live born children, and 13,988 of these children were alive at 1 year of age and subsequently were followed up. The cohort has been followed since birth with annual questionnaires and, since 7 years of age, with objective measures in annual research clinics. The study protocol has been described previously,^30^, ^31^ and further information can be found at http://www.alspac.bris.ac.uk. Ethics approval for all aspects of data collection was obtained from the ALSPAC Ethics and Law Committee (IRB 00003312) and the Local Research Ethics Committees.

### Exposures

Mothers were asked 8 weeks into their pregnancy to report their current alcohol consumption (how many measures of beer, wine, spirit, or other alcoholic drinks per day on weekdays and weekends), from which the total units/week were derived and then grouped to avoid categories with small numbers (never, 1-2 units/week, 3-4 units/week, ≥5 units/week). At 18 weeks of gestation mothers were asked to recall their alcohol consumption just before the current pregnancy (derived categories: never, <1 unit/week, 1-6 units/week, ≥7 units/week) and in the first 3 months of pregnancy (never, <1 unit/week, >1 unit/week). (Asking about drinking habits just before pregnancy is likely to capture intake in early gestation, before mothers have realized that they have conceived and had a chance to alter their drinking behavior). At 8 weeks after delivery mothers were asked to report their consumption in the last 2 months of pregnancy (never, <1 unit/week, >1 unit/week). We also defined mothers as “binge” drinkers if they reported drinking at least 4 units on 1 or more days (never, 1-2 days, ≥3 days) in the previous month when asked at 18 weeks of gestation.^28^

### Outcomes

When the children were 7.5 years old, mothers were asked, “Has your child had any of the following in the past 12 months: wheezing, asthma, eczema, hay fever?” Children were defined as having current doctor-diagnosed asthma at 7.5 years (primary outcome of interest) if mothers responded positively to the question, “Has a doctor *ever* actually *said* that your study child has asthma?” *and* positively to 1 or both of the questions on wheezing and asthma in the past 12 months.

Atopy at 7 years was defined as a positive reaction (any detectable weal) to *Dermatophagoides pteronyssinus*, cat, or grass (after subtracting positive saline reactions from histamine and allergen wheals, and excluding children unreactive to 1% histamine). (Atopy defined in this way identified 96% of children sensitized to 26 other allergens in this cohort.^32^) Serum total IgE (in kU/L) was measured by fluoroimmunoassay with the Pharmacia UNICAP system (Pharmacia & Upjohn Diagnostics AB, Uppsala, Sweden).

### Confounders

In multivariate analyses we controlled for the following potential confounders: maternal factors during pregnancy (smoking, infections, anxiety score, antibiotic use, and paracetamol use); other maternal factors (educational level; housing tenure; financial difficulties; body mass index; age; parity; history of asthma, eczema, rhinoconjunctivitis, migraine); sex of child, season of birth, multiple pregnancy, gestational age, birth weight, head circumference, birth length; postnatal factors (breast-feeding, day care, pets, damp/mold, environmental tobacco smoke exposure, antibiotic and paracetamol use in infancy, number of younger siblings, body mass index at age 7).

### Genotyping

The majority of maternal DNA samples were extracted from whole blood and white cells taken during pregnancy, and a minority were buccal DNA extracted from mouthwash samples. A single nucleotide polymorphism (rs1229984 in *ADH1B*) was typed by KBiosciences Ltd (Hoddesdon, Herts, United Kingdom; www.kbioscience.co.uk) with a competitive allele-specific PCR system (KASPar). The genotype distribution was compatible with no departure from Hardy-Weinberg equilibrium, and the genotyping success rate was >93.3% and error rate <0.25%.^28^

### Statistical analyses

Although the ALSPAC population is largely white, we adopted 2 strategies to reduce the possibility of confounding by population substructure, because the rs1229984 variant is highly ethnically stratified. First, mother-child pairs were excluded from all analyses if the mother’s reported ethnicity was non-white or unknown. Second, to address possible residual confounding by population substructure, we controlled for 10 variables derived by principal components analysis (PCA) from ALSPAC genome-wide association study (GWAS) data.

We used logistic regression to analyze relations of maternal *ADH1B* genotype with binary outcomes and linear regression to analyze associations with log-transformed total IgE. Analyses of reported alcohol intake in pregnancy were performed before and after controlling for confounders; tests for trend were performed by analyzing reported alcohol exposure associations as linear per category effects. In view of the rarity of the homozygous mutant genotype (A:A), persons with this genotype were grouped together with heterozygotes (G:A), and we assumed a dominant effect for analysis. Previous analyses of this single nucleotide polymorphism in ALSPAC mothers in relation to alcohol intake suggested that this was appropriate.^28^ We also performed *a priori* stratified analyses to explore potential interactions between maternal *ADH1B* genotype and reported alcohol intake in pregnancy. All analyses were performed with Stata version 10.1 (Stata Corporation, College Station, Tex).

## Results

Table I shows the association between reported alcohol intake in the last 2 months of pregnancy and other maternal characteristics among mothers for whom data were complete for at least 1 childhood atopic outcome. Mothers who reported drinking alcohol were more likely than mothers who reported never drinking to be older, better educated, financially better off, and to have taken paracetamol in pregnancy. In contrast, Table II shows that the distribution of maternal *ADH1B* genotype broadly does not vary according to maternal or offspring characteristics.

**Table I:** Maternal characteristics by alcohol consumption in the last 2 months of pregnancy

	Alcohol consumption (units/wk)		
	Never, no. (%) (N = 3780)	<1, no. (%) (N = 2947)	≥1, no. (%) (N = 1475)
Mother’s age			
<20 y	108 (3)	40 (1)	9 (1)
20-24 y	684 (18)	338 (11)	81 (5)
25-29 y	1564 (41)	1258 (43)	466 (32)
30-34 y	1077 (28)	983 (33)	630 (43)
≥35 y	347 (9)	328 (11)	289 (20)
Parity			
0	1727 (46)	1284 (44)	619 (42)
1	1255 (33)	1095 (37)	527 (36)
≥2	716 (19)	510 (17)	299 (20)
Unknown	82 (2)	58 (2)	30 (2)
Maternal body mass index (kg/m^2^)			
<18.5	171 (5)	109 (4)	54 (4)
18.5-24.99	2527 (67)	2108 (72)	1083 (73)
25-29.99	549 (15)	403 (14)	188 (13)
≥30	217 (6)	116 (4)	53 (4)
Unknown	316 (8)	211 (7)	97 (7)
Mother’s education level			
<Ordinary level	1098 (29)	558 (19)	225 (15)
Ordinary level	1468 (39)	1089 (37)	387 (26)
≥Advanced level	1198 (32)	1292 (44)	861 (58)
Unknown	16 (<1)	8 (<1)	2 (<1)
Housing tenure			
Owned/mortgaged	2915 (77)	2477 (84)	1261 (85)
Rented (public housing)	436 (12)	194 (7)	64 (4)
Rented (nonpublic housing)	257 (7)	152 (5)	99 (7)
Unknown/other	172 (5)	124 (4)	51 (3)
Financial difficulties			
None	1425 (38)	1111 (38)	657 (45)
Some	1337 (35)	1144 (39)	499 (34)
Many	904 (24)	627 (21)	279 (19)
Unknown	114 (3)	65 (2)	40 (3)
Maternal asthma	246 (7)	175 (6)	81 (5)
Maternal eczema	422 (11)	358 (12)	179 (12)
Maternal rhinoconjunctivitis	665 (18)	585 (20)	292 (20)
Maternal migraine	577 (15)	398 (14)	188 (13)
Maternal anxiety score in pregnancy			
0-4	1920 (51)	1514 (51)	757 (51)
5-9	1218 (32)	951 (32)	468 (32)
≥10	335 (9)	269 (9)	145 (10)
Unknown	307 (8)	213 (7)	105 (7)
Prenatal tobacco exposure (maximum in pregnancy)			
Not exposed	1659 (44)	1436 (49)	695 (47)
Passive only	1267 (34)	967 (33)	472 (32)
Mother 1-9/d	283 (7)	225 (8)	145 (10)
Mother 10-19/d	395 (10)	211 (7)	101 (7)
Mother ≥20/d	158 (4)	97 (3)	54 (4)
Unknown	18 (<1)	11 (<1)	8 (1)
Maternal paracetamol use in early pregnancy			
Nonuser	1873 (50)	1260 (43)	600 (41)
User	1850 (49)	1654 (56)	857 (58)
Unknown	57 (2)	33 (1)	18 (1)
Maternal paracetamol use in late pregnancy			
Nonuser	2219 (59)	1580 (54)	774 (52)
User	1447 (38)	1296 (44)	662 (45)
Unknown	114 (3)	71 (2)	39 (3)
Antibiotic use in late pregnancy	203 (5)	209 (7)	93 (6)
Maternal cold/flu in pregnancy	1478 (39)	1230 (42)	659 (45)
Maternal urinary infection in pregnancy	198 (5)	157 (5)	79 (5)
Maternal other infections in pregnancy	185 (5)	191 (6)	97 (7)

Includes all women with complete data for at least 1 childhood outcome (wheeze, eczema, hay fever, asthma, atopy, IgE) and self-reported alcohol consumption in the last 2 months of pregnancy.

**Table II:** Maternal characteristics by maternal *ADH1B* genotype

	*ADH1B* genotype	
	GG, no. (%) (N = 5033)	AA/GA, no. (%) (N = 268)
Mother’s age		
<20 y	86 (2)	3 (1)
20-24 y	671 (13)	37 (14)
25-29 y	2056 (41)	105 (39)
30-34 y	1657 (33)	93 (35)
≥35 y	563 (11)	30 (11)
Parity		
0	2275 (45)	118 (44)
1	1750 (35)	92 (34)
≥2	909 (18)	52 (19)
Unknown	99 (2)	6 (2)
Maternal body mass index (kg/m^2^)		
<18.5	208 (4)	14 (5)
18.5-24.99	3460 (69)	192 (72)
25-29.99	726 (14)	35 (13)
≥30	248 (5)	9 (3)
Unknown	391 (8)	18 (7)
Mother’s education level		
<Ordinary level	1186 (24)	47 (18)
Ordinary level	1811 (36)	108 (40)
≥Advanced level	2023 (40)	113 (42)
Unknown	13 (<1)	0 (0)
Housing tenure		
Owned/mortgaged	4093 (81)	222 (83)
Rented (public housing)	439 (9)	23 (9)
Rented (nonpublic housing)	297 (6)	16 (6)
Unknown/other	204 (4)	7 (3)
Financial difficulties		
None	1957 (39)	104 (39)
Some	1816 (36)	93 (35)
Many	1127 (22)	66 (25)
Unknown	133 (3)	5 (2)
Maternal asthma	314 (6)	16 (6)
Maternal eczema	588 (12)	32 (12)
Maternal rhinoconjunctivitis	970 (19)	60 (22)
Maternal migraine	729 (14)	38 (14)
Maternal anxiety score in pregnancy		
0-4	2664 (53)	133 (50)
5-9	1594 (32)	94 (35)
≥10	462 (9)	32 (12)
Unknown	313 (6)	9 (3)
Prenatal tobacco exposure (maximum in pregnancy)		
Not exposed	2285 (45)	133 (50)
Passive only	1700 (34)	94 (35)
Mother 1-9/d	405 (8)	13 (5)
Mother 10-19/d	429 (9)	19 (7)
Mother ≥20/d	191 (4)	9 (3)
Unknown	23 (<1)	0 (0)
Maternal paracetamol use in early pregnancy		
Non user	2267 (45)	132 (49)
User	2691 (53)	133 (50)
Unknown	75 (1)	3 (1)
Maternal paracetamol use in late pregnancy		
Non user	2785 (55)	158 (59)
User	2113 (42)	104 (39)
Unknown	135 (3)	6 (2)
Antibiotic use in late pregnancy	315 (6)	17 (6)
Maternal cold/flu in pregnancy	2060 (41)	105 (39)
Maternal urinary infection in pregnancy	274 (5)	10 (4)
Maternal other infections in pregnancy	292 (6)	12 (4)

Includes all women with complete data for at least 1 childhood outcome and *ADH1B* genotype.

Table E1 (in the Online Repository at www.jacionline.org) shows the association between maternal ADH1B genotype and reported maternal alcohol intake before and during pregnancy. As expected, carriers of the A allele reported a lower intake than those homozygous for the G allele both before and during pregnancy, they were less likely to report binge drinking, and they were more likely to report abstaining from alcohol during pregnancy.

Table III shows the association between self-reported alcohol consumption before and during pregnancy and childhood asthma in persons with complete outcome data. Univariately, evidence was weak for a lower prevalence of asthma in children of mothers who reported consuming alcohol in the last 2 months of pregnancy. After controlling for confounders, evidence was still weak for a lower prevalence of asthma in mothers who reported consuming alcohol in the first 3 months (test for trend, *P* = .047) and last 2 months of pregnancy (test for trend, *P* = .074). Table IV shows that, after controlling for confounders, reported maternal alcohol intake in late pregnancy was negatively associated with hay fever in the offspring (test for trend, *P* = .024). Table E2, Table E3, Table E4, Table E5 (in the Online Repository at www.jacionline.org) show the associations between reported alcohol consumption before and during pregnancy and childhood wheezing, eczema, atopy, and IgE. After controlling for confounders, maternal intake was not associated with any outcome.

**Table III:** Associations between self-reported alcohol consumption before and during pregnancy and childhood asthma

	Univariate analysis, OR (95% CI)	Multivariate analysis, OR (95% CI)
Mother's alcohol intake before pregnancy (units/wk)		
Never (n = 446)	1.0	1.0
<1 (n = 2860)	0.88 (0.66-1.18)	0.93 (0.69-1.26)
1-6 (n = 3410)	0.85 (0.64-1.14)	0.92 (0.68-1.25)
≥7 (n = 890)	0.81 (0.57-1.14)	0.84 (0.59-1.21)
Test for trend (N = 7606)	0.95 (0.86-1.04)∗	0.96 (0.87-1.05)∗
*P* value	.229	.383
Mother’s current alcohol intake at 8 wk of pregnancy (units/wk)		
Never (n = 4728)	1.0	1.0
1-2 (n = 973)	0.97 (0.78-1.20)	0.96 (0.77-1.20)
3-4 (n = 524)	1.06 (0.81-1.39)	1.03 (0.78-1.37)
≥5 (n = 659)	1.02 (0.79-1.30)	0.93 (0.71-1.20)
Test for trend (N = 6884)	1.00 (0.94-1.09)∗	0.98 (0.91-1.06)∗
*P* value	.808	.667
Mother’s alcohol intake in first 3 mo of pregnancy (units/wk)		
Never (n = 3356)	1.0	1.0
<1 (n = 3088)	0.96 (0.82-1.11)	0.91 (0.78-1.06)
≥1 (n = 1148)	0.89 (0.72-1.09)	0.81 (0.65-1.00)
Test for trend (N = 7592)	0.95 (0.86-1.04)∗	0.90 (0.81-0.99)∗
*P* value	.262	.047
Mother’s alcohol intake during last 2 mo of pregnancy (units/wk)		
Never (n = 3410)	1.0	1.0
<1 (n = 2668)	0.92 (0.79-1.08)	0.92 (0.78-1.08)
≥1 (n = 1327)	0.83 (0.68-1.02)	0.83 (0.67-1.02)
Test for trend (N = 7405)	0.91 (0.83-1.00)∗	0.91 (0.82-1.01)∗
*P* value	.062	.074
Mother’s binge drinking (days in previous month at 18 wk of pregnancy)		
Never (n = 6358)	1.0	1.0
1-2 (n = 630)	1.11 (0.87-1.42)	0.95 (0.74-1.23)
≥3 (n = 516)	0.98 (0.75-1.30)	0.84 (0.63-1.12)
Test for trend (N = 7504)	1.02 (0.90-1.15)∗	0.92 (0.81-1.05)∗
*P* value	.783	.235

*OR*, Odds ratio.

∗ OR per category increase in exposure. Multivariate analysis of maternal alcohol intake adjusts for all confounders listed in Methods section.

**Table IV:** Associations between self-reported alcohol consumption before and during pregnancy and childhood hay fever

	Univariate analysis, OR (95% CI)	Multivariate analysis. OR (95% CI)
Mother's alcohol intake before pregnancy (units/wk)		
Never (n = 449)	1.0	1.0
<1 (n = 2875)	1.28 (0.87-1.87)	1.27 (0.86-1.89)
1-6 (n = 3428)	1.26 (0.87-1.84)	1.17 (0.79-1.73)
≥7 (n = 903)	1.15 (0.74-1.77)	1.03 (0.66-1.62)
Test of trend (N = 7655)	1.0 (0.90-1.11)∗	0.96 (0.86-1.07)∗
*P* value	.944	.437
Mother’s current alcohol intake at 8 wk of pregnancy (units/wk)		
Never (n = 4753)	1.0	1.0
1-2 (n = 977)	1.02 (0.81-1.30)	0.95 (0.74-1.22)
3-4 (n = 530)	1.02 (0.74-1.39)	1.04 (0.75-1.44)
≥5 (n = 670)	0.70 (0.51-0.97)	0.71 (0.50-0.99)
Test for trend (N = 6930)	0.93 (0.85-1.01)∗	0.93 (0.84-1.02)∗
*P* value	.095	.109
Mother’s alcohol intake in first 3 mo of pregnancy (units/wk)		
Never (n = 3370)	1.0	1.0
<1 (n = 3100)	0.83 (0.70-0.99)	0.84 (0.70-1.01)
≥1 (n = 1172)	0.88 (0.70-1.11)	0.91 (0.71-1.17)
Test for trend (N = 7642)	0.91 (0.81-1.02)∗	0.93 (0.82-1.04)∗
*P* value	.103	.203
Mother’s alcohol intake during last 2 mo of pregnancy (units/wk)		
Never (n = 3434)	1.0	1.0
<1 (n = 2678)	0.92 (0.77-1.09)	0.85 (0.71-1.03)
≥1 (n = 1346)	0.83 (0.66-1.05)	0.77 (0.60-0.99)
Test for trend (N = 7458)	0.91 (0.82-1.02)∗	0.87 (0.78-0.98)∗
*P* value	.101	.024
Mother’s binge drinking (days in previous month at 18 wk of pregnancy)		
None (n = 6396)	1.0	1.0
1-2 (n = 633)	0.91 (0.68-1.23)	0.96 (0.71-1.31)
≥3 (n = 523)	0.73 (0.51-1.04)	0.78 (0.54-1.13)
Test for trend (N = 7552)	0.87 (0.74-1.02)∗	0.90 (0.77-1.06)∗
*P* value	.079	.219

*OR*, Odds ratio.

∗ OR per category increase in exposure. Multivariate analysis of maternal alcohol intake adjusts for all confounders listed in Methods section.

Table V shows the associations between maternal *ADH1B* genotype and childhood atopic outcomes. Univariately, no association was observed with any outcome. Similarly, among persons with available PCA variables derived from GWAS data, maternal genotype was not associated with any outcome, either univariately or after controlling for all potential confounders, including the 10 PCA variables.

**Table V:** Associations between maternal *ADH1B* genotype and childhood atopic outcomes

Outcome by *ADH1B* genotype	No.	Univariate maternal effect estimate∗ (95% CI)	No. of reduced data set†	Univariate maternal effect estimate∗ (95% CI)	Multivariate maternal effect estimate∗ (95% CI)
Asthma					
G:G	4524	1.0	3266	1.0	1.0
A:A/G:A	231	0.98 (0.66-1.47)	171	0.86 (0.53-1.41)	0.90 (0.54-1.51)
Total	4755		3437		
*P* value		.937		.571	.699
Wheezing					
G:G	4575	1.0	3280	1.0	1.0
A:A/G:A	237	1.12 (0.75-1.67)	171	1.04 (0.64-1.68)	1.08 (0.65-1.79)
Total	4812		3451		
*P* value		.582		.867	.754
Eczema					
G:G	4567	1.0	3295	1.0	1.0
A:A/G:A	234	1.01 (0.71-1.43)	170	1.07 (0.72-1.60)	1.14 (0.75-1.73)
Total	4801		3465		
*P* value		.961		.730	.539
Hay fever					
G:G	4556	1.0	3248	1.0	1.0
A:A/G:A	236	1.11 (0.71-1.72)	171	1.12 (0.67-1.87)	1.15 (0.67-1.97)
Total	4792		3419		
*P* value		.653		.673	.616
Atopy					
G:G	3571	1.0	2811	1.0	1.0
A:A/G:A	193	1.09 (0.77-1.55)	152	1.12 (0.76-1.65)	1.23 (0.82-1.84)
Total	3764		2963		
*P* value		.610		.571	.327
IgE					
G:G	2852	1.0	2404	1.0	1.0
A:A/G:A	158	1.07 (0.82-1.40)	133	0.97 (0.72-1.30)	0.96 (0.72-1.30)
Total	3010		2537		
*P* value		.606		.815	.806

*OR*, Odds ratio.

∗ OR (geometric mean ratio for IgE analysis).

† Data set reduced because of incomplete PCA variables derived from GWAS data.

No evidence suggests that reported maternal alcohol intake in late pregnancy (comparing 1 or more units per week with never) modified the associations between maternal *ADH1B* genotype and childhood asthma and other atopic outcomes (Table VI), and no interactions were found with reported alcohol intake during early pregnancy (data not shown).

**Table VI:** Associations between maternal *ADH1B* genotype and childhood atopic outcomes stratified by reported alcohol intake during the last 2 mo of pregnancy

Outcome by *ADH1B* genotype	Mother’s alcohol intake (units/wk) never		Mother’s alcohol intake (units/wk) ≥1	
	No.	Adjusted estimate∗ (95% CI)	No.	Adjusted estimate∗ (95% CI)
Asthma†				
G:G	2001	1.0	2372	1.0
G:A/A:A	121	0.94 (0.53-1.68)	103	1.14 (0.62-2.10)
Total	4597			
Wheezing‡				
G:G	2024	1.0	2400	1.0
G:A/A:A	124	1.26 (0.72-2.19)	105	0.87 (0.43-1.73)
Total	4653			
Eczema§				
G:G	2018	1.0	2397	1.0
G:A/A:A	121	0.89 (0.52-1.52)	105	1.23 (0.75-2.02)
Total	4641			
Hay fever‖				
G:G	2017	1.0	2388	1.0
G:A/A:A	124	0.98 (0.52-1.87)	104	1.23 (0.62-2.46)
Total	4633			
Atopy¶				
G:G	1538	1.0	1894	1.0
G:A/A:A	102	1.29 (0.80-2.09)	83	1.12 (0.65-1.95)
Total	3617			
IgE∗∗				
G:G	1191	1.0	1557	1.0
G:A/A:A	85	1.23 (0.85-1.78)	70	0.91 (0.61-1.36)
Total	2903			

*GMR*, Geometric mean ratio.

∗ Estimates (OR [GMR for IgE analysis]) are adjusted for all confounders listed in Methods section.

† *P* for interaction = .664.

‡ *P* for interaction = .411.

§ *P* for interaction = .384.

‖ *P* for interaction = .636.

¶ *P* for interaction = .710.

∗∗ *P* for interaction = .288.

## Discussion

In this population-based birth cohort we have shown, using a Mendelian randomization approach, that a maternal *ADH1B* gene variant (rs1229984), as a proxy for prenatal alcohol exposure, was unrelated to childhood atopic outcomes, suggesting that alcohol consumption during pregnancy is unlikely to be an important cause of these disorders. This conclusion is further reinforced by the lack of evidence for an interaction between maternal *ADH1B* and reported intake of alcohol in pregnancy; such an interaction, with a weaker or absent effect of the gene in mothers reporting no alcohol intake, would have been expected if there was a causal effect of prenatal alcohol exposure.^33^ The lack of evidence of association between maternal *ADH1B* genotype and atopic outcomes in the offspring also suggests that maternal consumption of alcohol before, or periconception, is unlikely to influence childhood asthma and allergies; in ALSPAC the same nonsynonymous variant of *ADH1B* is strongly associated with alcohol use just before, as well as during, pregnancy.^28^

Given the principles underpinning Mendelian randomization,^34^, ^35^ our genetic findings are much less likely to be confounded by lifestyle or environmental factors than the weak negative associations we observed between reported maternal alcohol intake in late pregnancy and risk of asthma and hay fever in the offspring. Mothers who drank alcohol in pregnancy tended to be older, better educated, and financially better off than mothers who abstained. Although we controlled for a large number of socioeconomic and other background characteristics in the analyses of reported maternal drinking, the possibility of residual or uncontrolled confounding remains. Furthermore, the negative associations between reported maternal intake and childhood asthma and hay fever conflict with the few previous epidemiologic studies that have suggested a positive relation between prenatal alcohol exposure and atopic outcomes.^11^, ^12^, ^13^ Although there is some evidence for a protective effect of alcohol on allergic airway inflammation and airway hyperresponsiveness in an adult mouse model of allergic asthma,^3^ the mechanisms underlying such an effect and the relevance of these observations to the inception of asthma after prenatal exposure in humans are unclear. In addition, other animal data indicate detrimental effects of prenatal exposure on fetal lung growth and development.^4^, ^5^, ^6^ The findings for hay fever, if real, would be expected to be mediated through an effect on atopy, yet we did not find similar negative associations between reported intake and atopy or IgE. We therefore think it is unlikely that prenatal alcohol exposure in pregnancy protects against asthma and other atopic disease.

In ALSPAC, mothers carrying the minor A allele consumed less alcohol during pregnancy. Previous *in vitro* data suggested that carriers of this allele metabolize alcohol up to 100 times faster than persons homozygous for the G allele^36^ and consequently consume less alcohol to avoid the negative effects of high acetaldehyde concentrations; this in turn would reduce exposure of the fetus to alcohol, which readily crosses the placenta. Although a more recent study has suggested that this variant makes a minor contribution to the variation in alcohol metabolism *in vivo,*^37^ lack of power because of the variant’s low frequency in European populations is the most likely explanation for this. The utility of *ADH1B* genotype as a Mendelian randomization instrument has been clearly demonstrated in relation to alcohol-induced cancer.^27^, ^33^

### Strengths and limitations

Our study has a number of strengths, including ALSPAC’s population-based prospective design and rich data on maternal reported alcohol use in pregnancy, potential confounders and detailed phenotypic outcome measurements. In addition, few other birth cohorts have maternal DNA available. Measurement of maternal *ADH1B* genotype enabled us to use a Mendelian randomization approach to investigate the relation between prenatal alcohol exposure and atopic outcomes. In contrast, most previous epidemiologic studies of the effects of maternal alcohol use in pregnancy on offspring health have relied on reported consumption, an approach which is highly susceptible to bias and confounding. Although there was potential for associations with *ADH1B* genotype to be confounded by population substructure, we reduced this possibility by excluding those with reported non-white ethnicity and by performing sensitivity analyses in which we controlled for PCA variables derived from GWAS data. We acknowledge, however, that, given the low minor allele frequency for rs1229984, we lacked power to detect small or modest effects of this variant, as indicated by the confidence intervals around the effect estimates, and also power to detect interactions with reported alcohol intake. We also accept that we have not taken into account the ability of the fetus to metabolize alcohol and hence the potential effect of gene variants in the child on atopic outcomes. Nevertheless, we would argue that the maternal *ADH1B* gene is likely to have a greater influence on fetal alcohol exposure because it is so strongly associated with maternal drinking behavior. As with any longitudinal study, data were not complete on exposures, outcomes, and confounders for the whole cohort. Therefore, we cannot rule out the possibility that exclusion of children without complete information might have biased our findings for reported alcohol intake; however, importantly, it is unlikely that missing genotype data would bias the main genetic results.

### Public health implications

In conclusion, with the use of a Mendelian randomization approach, we have not found evidence to suggest that alcohol consumption in pregnancy increases the risk of childhood atopic disease. Nor did associations with reported intake suggest increased risks. Nevertheless, because of other potential risks to the developing fetus of low-level alcohol consumption, pregnant women should still heed current advice to abstain from consuming alcohol.^38^, ^39^

**Clinical implications**

Although pregnant women should be advised to abstain from alcohol, this study has reassuringly found no evidence that alcohol exposure *in utero* increases the risk of atopic disease in the offspring.

## Figures and Tables

**Table E1:** Association between maternal *ADH1B* genotype and reported maternal alcohol intake before and during pregnancy

	*ADH1B* genotype	
	GG, No. (%) (N = 5033)	GA/AA, No. (%) (N = 268)
Mother’s alcohol intake before pregnancy (units/wk)		
Never	275 (5)	18 (7)
<1	1820 (36)	126 (47)
1-6	2273 (45)	102 (38)
≥7	599 (12)	20 (7)
Unknown	66 (1)	2 (1)
Mother’s current alcohol intake at 8 wk of pregnancy (units/wk)		
Never	3074 (61)	197 (74)
1-2	686 (14)	30 (11)
3-4	342 (7)	17 (6)
≥5	454 (9)	9 (3)
Unknown	477 (9)	15 (6)
Mother’s alcohol intake in first 3 mo of pregnancy (units/wk)		
Never	2130 (42)	138 (51)
<1	2035 (40)	99 (37)
≥1	795 (16)	27 (10)
Unknown	73 (1)	4 (1)
Mother’s alcohol intake during last 2 mo of pregnancy (units/wk)		
Never	2211 (44)	143 (53)
<1	1722 (34)	81 (30)
≥1	898 (18)	34 (13)
Unknown	202 (4)	10 (4)
Mother’s binge drinking (days in previous month at 18 wk of pregnancy)		
None	4164 (83)	237 (88)
1-2	441 (9)	14 (5)
≥3	351 (7)	13 (5)
Unknown	77 (2)	4 (1)

Includes all women with complete data for at least 1 childhood outcome and ADH1B genotype.

**Table E2:** Associations between self-reported alcohol consumption before and during pregnancy and childhood wheezing

	Univariate analysis, OR (95% CI)	Multivariate analysis, OR (95% CI)
Mother’s alcohol intake before pregnancy (units/wk)		
Never (n = 452)	1.0	1.0
<1 (n = 2888)	0.91 (0.66-1.24)	0.96 (0.69-1.32)
1-6 (n = 3443)	0.88 (0.65-1.20)	0.92 (0.67-1.28)
≥7 (n = 904)	0.96 (0.67-1.37)	0.95 (0.65-1.38)
Test for trend (N = 7687)	0.99 (0.90-1.09)	0.98 (0.89-1.08)
*P* value	.820	.703
Mother’s current alcohol intake at 8 wk of pregnancy (units/wk)		
Never (n = 4779)	1.0	1.0
1-2 (n = 981)	1.08 (0.87-1.34)	1.03 (0.82-1.30)
3-4 (n = 530)	1.12 (0.85-1.49)	1.08 (0.80-1.44)
≥5 (n = 671)	1.05 (0.81-1.37)	0.95 (0.73-1.25)
Test for trend (N = 6961)	1.03 (0.96-1.11)∗	0.99 (0.92-1.08)∗
*P* value	.439	.957
Mother’s alcohol intake in first 3 mo of pregnancy (units/wk)		
Never (n = 3386)	1.0	1.0
<1 (n = 3113)	1.04 (0.89-1.22)	0.98 (0.83-1.16)
≥1 (n = 1174)	1.05 (0.85-1.30)	0.95 (0.76-1.19)
Test for trend (N = 7673)	1.03 (0.93-1.14)∗	0.98 (0.88-1.09)∗
*P* value	.575	.664
Mother’s alcohol intake during last 2 mo of pregnancy (units/wk)		
Never (n = 3445)	1.0	1.0
<1 (n = 2696)	0.94 (0.80-1.11)	0.91 (0.76-1.08)
≥1 (n = 1346)	0.98 (0.80-1.20)	0.92 (0.74-1.14)
Test for trend (N = 7487)	0.98 (0.89-1.08)∗	0.95 (0.85-1.06)∗
*P* value	.706	.335
Mother’s binge drinking (days in previous month at 18 wk of pregnancy)		
None (n = 6423)	1.0	1.0
1-2 (n = 637)	0.97 (0.74-1.27)	0.85 (0.64-1.13)
≥3 (n = 523)	1.12 (0.85-1.49)	0.99 (0.74-1.33)
Test for trend (N = 7583)	1.04 (0.92-1.18)∗	0.97 (0.84-1.11)∗
*P* value	.536	.619

*OR*, Odds ratio.

∗ OR per category increase in exposure. Multivariate analysis of maternal alcohol intake adjusts for all confounders listed in Methods section.

**Table E3:** Associations between self-reported alcohol consumption before and during pregnancy and childhood eczema

	Univariate analysis, OR (95% CI)	Multivariate analysis, OR (95% CI)
Mother’s alcohol intake before pregnancy (units/wk)		
Never (n = 451)	1.0	1.0
<1 (n = 2880)	1.08 (0.82-1.43)	1.03 (0.77-1.36)
1-6 (n = 3438)	1.11 (0.85-1.46)	1.03 (0.78-1.37)
≥7 (n = 904)	1.13 (0.83-1.54)	0.99 (0.72-1.38)
Test for trend (N = 7673)	1.03 (0.95-1.12)	0.99 (0.92-1.09)
*P* value	.438	.970
Mother’s current alcohol intake at 8 wk of pregnancy (units/wk)		
Never (n = 4766)	1.0	1.0
1-2 (n = 980)	1.07 (0.89-1.29)	1.04 (0.86-1.26)
3-4 (n = 530)	1.00 (0.79-1.28)	1.02 (0.79-1.32)
≥5 (n = 670)	1.13 (0.91-1.40)	1.12 (0.90-1.40)
Test for trend (N = 6946)	1.03 (0.97-1.10)∗	1.03 (0.97-1.10)∗
*P* value	.303	.343
Mother’s alcohol intake in first 3 mo of pregnancy (units/wk)		
Never (n = 3379)	1.0	1.0
<1 (n = 3107)	0.99 (0.87-1.13)	0.98 (0.86-1.13)
≥1 (n = 1174)	1.06 (0.89-1.26)	1.08 (0.90-1.31)
Test for trend (N = 7660)	1.02 (0.94-1.11)∗	1.03 (0.94-1.12)∗
*P* value	.649	.554
Mother’s alcohol intake during last 2 mo of pregnancy (units/wk)		
Never (n = 3436)	1.0	1.0
<1 (n = 2692)	1.03 (0.90-1.18)	0.98 (0.85-1.13)
≥1 (n = 1346)	1.18 (0.99-1.39)	1.11 (0.93-1.33)
Test for trend (N = 7474)	1.08 (0.99-1.17)∗	1.04 (0.95-1.14)∗
*P* value	.077	.356
Mother’s binge drinking (days in previous month at 18 wk of pregnancy)		
None (n = 6411)	1.0	1.0
1-2 (n = 637)	0.84 (0.66-1.05)	0.84 (0.66-1.07)
≥3 (n = 522)	0.92 (0.72-1.18)	0.93 (0.72-1.20)
Test for trend (N = 7570)	0.93 (0.83-1.04)∗	0.94 (0.83-1.06)∗
*P* value	.227	.297

*OR*, Odds ratio.

∗ OR per category increase in exposure. Multivariate analysis of maternal alcohol intake adjusts for all confounders listed in Methods section.

**Table E4:** Associations between self-reported alcohol consumption before and during pregnancy and childhood atopy

	Univariate analysis, OR (95% CI)	Multivariate analysis, OR (95% CI)
Mothers alcohol intake before pregnancy (units/wk)		
Never (n = 330)	1.0	1.0
<1 (n = 2287)	1.12 (0.83-1.51)	1.09 (0.80-1.48)
1-6 (n = 2717)	1.21 (0.90-1.62)	1.08 (0.80-1.47)
≥7 (n = 725)	1.40 (1.01-1.94)	1.22 (0.87-1.73)
Test of trend (N = 6059)	1.11 (1.02-1.20)∗	1.05 (0.96-1.14)∗
*P* value	.014	.304
Mother’s current alcohol intake at 8 wk of pregnancy (units/wk)		
Never (n = 3713)	1.0	1.0
1-2 (n = 780)	1.07 (0.89-1.29)	1.05 (0.86-1.27)
3-4 (n = 427)	0.99 (0.78-1.27)	1.01 (0.78-1.30)
≥5 (n = 525)	0.80 (0.63-1.01)	0.80 (0.63-1.03)
Test for trend (N = 5445)	0.95 (0.89-1.02)∗	0.95 (0.89-1.02)∗
*P* value	.167	.198
Mother’s alcohol intake in first 3 mo of pregnancy (units/wk)		
Never (n = 2614)	1.0	1.0
<1 (n = 2502)	0.98 (0.86-1.12)	1.00 (0.87-1.15)
≥1 (n = 939)	0.96 (0.80-1.15)	0.96 (0.79-1.17)
Test for trend (N = 6055)	0.98 (0.90-1.07)∗	0.99 (0.90-1.08)∗
*P* value	.639	.776
Mother’s alcohol intake during last 2 mo of pregnancy (units/wk)		
Never (n = 2615)	1.0	1.0
<1 (n = 2133)	1.04 (0.90-1.19)	1.01 (0.87-1.16)
≥1 (n = 1098)	0.99 (0.83-1.18)	0.92 (0.76-1.10)
Test for trend (N = 5846)	1.0 (0.92-1.09)∗	0.96 (0.88-1.05)∗
*P* value	.965	.429
Mother’s binge drinking (days in previous month at 18 wk of pregnancy)		
None (n = 5021)	1.0	1.0
1-2 (n = 527)	0.96 (0.77-1.20)	0.99 (0.78-1.24)
≥3 (n = 419)	0.80 (0.61-1.03)	0.86 (0.66-1.13)
Test for trend (N = 5967)	0.91 (0.81-1.02)∗	0.94 (0.83-1.06)∗
*P* value	.101	.319

*OR*, Odds ratio.

∗ OR per category increase in exposure. Multivariate analysis of maternal alcohol intake adjusts for all confounders listed in Methods section.

**Table E5:** Associations between self-reported alcohol consumption before and during pregnancy and childhood IgE

	Univariate analysis, GMR (95% CI)	Multivariate analysis, GMR (95% CI)
Mother’s alcohol intake before pregnancy (units/wk)		
Never (n = 254)	1.0	1.0
<1 (n = 1816)	1.07 (0.86-1.34)	1.11 (0.89-1.38)
1-6 (n = 2179)	1.09 (0.88-1.35)	1.16 (0.93-1.44)
≥7 (n = 554)	0.96 (0.75-1.23)	1.04 (0.81-1.34)
Test for trend (N = 4803)	0.98 (0.92-1.05)	1.01 (0.95-1.08)
*P* value	.602	.715
Mother’s current alcohol intake at 8 wk of pregnancy (units/wk)		
Never (n = 2920)	1.0	1.0
1-2 (n = 649)	1.0 (0.87-1.16)	1.01 (0.87-1.16)
3-4 (n = 345)	1.10 (0.91-1.32)	1.08 (0.90-1.30)
≥5 (n = 436)	1.08 (0.91-1.27)	1.06 (0.89-1.25)
Test for trend (N = 4350)	1.03 (0.98-1.08)∗	1.02 (0.97-1.08)∗
*P* value	.268	.390
Mother’s alcohol intake in first 3 mo of pregnancy (units/wk)		
Never (n = 2066)	1.0	1.0
<1 (n = 2002)	1.10 (0.99-1.22)	1.06 (0.96-1.18)
≥1 (n = 731)	1.09 (0.95-1.26)	1.06 (0.92-1.22)
Test for trend (N = 4799)	1.06 (0.99-1.13)∗	1.04 (0.97-1.11)∗
*P* value	.095	.306
Mother’s alcohol intake during last 2 mo of pregnancy (units/wk)		
Never (n = 2033)	1.0	1.0
<1 (n = 1730)	1.00 (0.90-1.12)	0.98 (0.88-1.10)
≥1 (n = 897)	1.08 (0.95-1.23)	1.05 (0.92-1.20)
Test for trend (N = 4660)	1.04 (0.97-1.10)∗	1.02 (0.95-1.09)∗
*P* value	.283	.584
Mother’s binge drinking (days in previous month at 18 wk of pregnancy)		
None (n = 3980)	1.0	1.0
1-2 (n = 426)	0.97 (0.82-1.15)	0.93 (0.79-1.10)
≥3 (n = 323)	0.93 (0.77-1.12)	0.89 (0.73-1.07)
Test for trend (N = 4729)	0.96 (0.89-1.05)∗	0.94 (0.86-1.02)∗
*P* value	.403	.158

*GMR*, Geometric mean ratio.

∗ GMR per category increase in exposure. Multivariate analysis of maternal alcohol intake adjusts for all confounders listed in Methods section.
